# Atypical periodic paralysis and myalgia: A novel *RYR1* phenotype

**DOI:** 10.1212/WNL.0000000000004894

**Published:** 2018-01-30

**Authors:** Emma Matthews, Christoph Neuwirth, Fatima Jaffer, Renata S. Scalco, Doreen Fialho, Matt Parton, Dipa Raja Rayan, Karen Suetterlin, Richa Sud, Roland Spiegel, Rachel Mein, Henry Houlden, Andrew Schaefer, Estelle Healy, Jacqueline Palace, Ros Quinlivan, Susan Treves, Janice L. Holton, Heinz Jungbluth, Michael G. Hanna

**Affiliations:** From the MRC Centre for Neuromuscular Diseases (E.M., F.J., R.S.S., D.F., M.P., D.R.R., K.S., H.H., E.H., R.Q., J.L.H., M.G.H.), Department of Molecular Neuroscience, UCL Institute of Neurology and National Hospital for Neurology and Neurosurgery, Queen Square, London, UK; Neuromuscular Diseases Unit/ALS Clinic (C.N.), Kantonsspital St. Gallen, Switzerland; Neurogenetics Unit (R.S., H.H.) and Department of Neuropathology (J.L.H.), National Hospital for Neurology and Neurosurgery, Queen Square, London, UK; Human Genetics Laboratory Genetica (R.S.), Zurich, Switzerland; Genetics Department (R.M.), Viapath, Guy's Hospital, London; Wellcome Trust Centre for Mitochondrial Research (A.S.), University of Newcastle, Framlington Place, Newcastle Upon Tyne, UK; Institute of Pathology (E.H.), Belfast Health and Social Care Trust, Northern Ireland; Department of Neurology (J.P.), John Radcliffe Hospital, Oxford, UK; Departments of Biomedicine and Anesthesia (S.T.), Basel University Hospital, Switzerland; Department of Life Sciences (S.T.), Microbiology and Applied Pathology Section, University of Ferrara, Italy; Department of Paediatric Neurology (H.J.), Neuromuscular Service, Evelina Children's Hospital, St. Thomas' Hospital; and Department of Basic and Clinical Neuroscience (H.J.), Institute of Psychiatry, Psychology and Neuroscience, and Randall Division of Cell and Molecular Biophysics (H.J.), Muscle Signalling Section, King's College, London, UK.

## Abstract

**Objective:**

To characterize the phenotype of patients with symptoms of periodic paralysis (PP) and ryanodine receptor (*RYR1*) gene mutations.

**Methods:**

Cases with a possible diagnosis of PP but additional clinicopathologic findings previously associated with *RYR1-*related disorders were referred for a tertiary neuromuscular clinical assessment in which they underwent detailed clinical evaluation, including neurophysiologic assessment, muscle biopsy, and muscle MRI. Genetic analysis with next-generation sequencing and/or targeted Sanger sequencing was performed.

**Results:**

Three cases with episodic muscle paralysis or weakness and additional findings compatible with a *RYR1*-related myopathy were identified. The McManis test, used in the diagnosis of PP, was positive in 2 of 3 cases. Genetic analysis of known PP genes was negative. *RYR1* analysis confirmed likely pathogenic variants in all 3 cases.

**Conclusions:**

*RYR1* mutations can cause late-onset atypical PP both with and without associated myopathy. Myalgia and cramps are prominent features. The McManis test may be a useful diagnostic tool to indicate *RYR1*-associated PP. We propose that clinicopathologic features suggestive of *RYR1*-related disorders should be sought in genetically undefined PP cases and that *RYR1* gene testing be considered in those in whom mutations in *SCN4A, CACNA1S*, and *KCNJ2* have already been excluded.

The skeletal muscle ryanodine receptor (*RYR1*) gene encodes the principal sarcoplasmic reticulum calcium release channel with a crucial role in excitation-contraction coupling. Mutations in *RYR1* are the most common genetic cause of nondystrophic neuromuscular disorders,^1,2^ associated with a wide spectrum of clinicopathologic features, ranging from various early-onset congenital myopathies—central core disease,^3^ multiminicore disease (MmD),^4^ centronuclear myopathy,^5^ and congenital fiber type disproportion^6^—to the malignant hyperthermia (MH) susceptibility trait. *RYR1* mutations may also give rise to episodic neuromuscular manifestations, including exertional myalgia and rhabdomyolysis,^7^ a late-onset axial myopathy,^8,9^ and have recently been associated with a novel bleeding disorder due to abnormal smooth muscle cell contractility.^10^ Episodes of atypical periodic paralysis (PP) have been previously reported in a single patient with a recessive *RYR1*-related myopathy,^11^ but it has been unclear whether this phenotype was unique to the reported individual or common across different *RYR1* genotypes. The association between *RYR1* and PP is not entirely unexpected, considering that dysfunction of Cav1.1, the other key player involved in excitation-contraction coupling and the principal *RYR1* interactor, is the most common cause of primary PP. Recently, both dominant and recessive families with a Cav1.1-related myopathy have been described,^12^ suggesting further phenotypic overlap between *RYR1* and Cav1.1 dysfunction and a continuum between myopathic and episodic phenotypes due to mutations in these genes. Here, we report 3 additional *RYR1*-mutated patients presenting with PP episodes and variable additional myopathic manifestations.

## Methods

We examined cases referred with a possible diagnosis of PP but additional clinicopathologic findings previously associated with *RYR1*-related disorders to the national referral center for skeletal muscle channelopathies in the United Kingdom and to the Swiss Neuromuscular Diseases Unit Center.

### Standard protocol approvals, registrations, and patient consents

All procedures were conducted as part of routine clinical care. The study was performed under the ethics guidelines issued by our institution, with written informed consent obtained from all participants for genetic studies.

Genetic analysis for PP genes *SCN4A*, *CACNA1S*, and *KCNJ2* was performed at the Neurogenetics Unit, National Hospital for Neurology and Neurosurgery as provided by the Channelopathy Highly Specialized National Service for rare disease. Samples underwent next-generation sequencing on an Illumina HiSeq after enrichment with an Illumina custom Nextera Rapid Capture panel (Illumina, Inc, San Diego, CA). For case 2, the library preparation and enrichment were performed with TruSight One kit (Illumina) according to protocol instructions, allowing analysis of a panel of ≈5,000 genes (including PP genes and *RYR1*). The library was quantified with the Qubit 2.0 Fluorometer system (Thermo Fisher Scientific, Waltham, MA), and 2 × 250-bp paired-end sequencing was performed on the MiSeq sequencer (Illumina), as well as sequences alignment (Burrows-Wheeler aligner) and variant calling (Genome Analysis Toolkit variant caller). The variants were then analyzed with VariantStudio (Illumina).

Additional targeted *RYR1* Sanger sequencing of all cases was performed at the Diagnostic DNA Laboratory at Guy's Hospital, London.

## Results

### Case 1

A 54-year-old man was born 7 weeks prematurely. An atrial septal defect was noted at birth and corrected at the age of 5 years. Walking was delayed until the age of 2 years. He was never able to run or to keep up with peers physically because of weakness of his arms and legs. In early childhood, he toe-walked, ultimately requiring Achilles tendon lengthening. Symptoms were effectively stable throughout childhood, but from the age of 20 years, there was slowly progressive proximal weakness.

**Figure 1:**
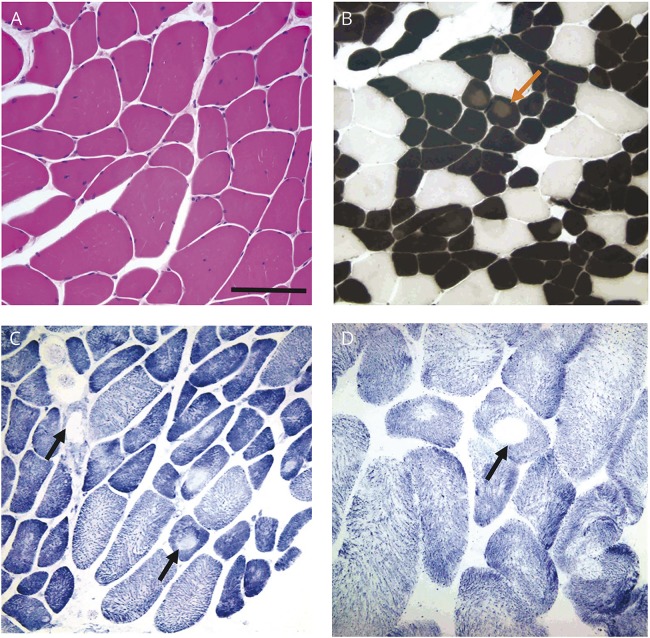
Histology slides of muscle biopsies taken from case 1 at age (A) 34 and (B) 42 years (A) Histologic examination of a right quadriceps muscle biopsy performed at age 34 years showed variation in fiber diameter and internalized nuclei with hematoxylin and eosin staining. (B) ATPase histochemistry at pH 4.3 indicated type I fiber predominance with decreased myofibrillar ATPase activity in core-like areas in up to 15% of type I fibers (red arrow). (C) Central absence of staining suggestive of cores was apparent in type I and II fibers in the nicotinamide dinucleotide tetrazolium reductase preparation (black arrows), and central reduction in oxidative enzyme activity was confirmed by succinate dehydrogenase staining (D, black arrow). Scale bar in A represents 100 μm in (A) and (C), 200 μm in (B), and 50 μm in (D).

At the age of 34 years, after a flu-like illness, he complained of a change in symptoms. He reported episodes of sudden severe myalgia followed by profound muscle weakness in either 1 limb or the entire body from the neck down lasting for several hours. Examination demonstrated a waddling gait and pronounced lumbar lordosis and mild dysmorphic features with a long thin face and high arched palate. Mild bilateral ptosis with a complex ophthalmoplegia most marked on upgaze, a typical finding in recessive *RYR1*-related myopathies, was noted. There was bilateral facial and sternocleidomastoid weakness with additional proximal upper and lower limb weakness. Reflexes were 1+, and there were no sensory abnormalities. Cardiac examination demonstrated atrial fibrillation for which he underwent cardioversion. Because of the change in symptoms, a muscle biopsy was performed. Although core-like structures were seen, they were not felt to be typical of central core disease, a common *RYR1*-related myopathy, and no definitive diagnosis was reached (figure 1).

From this point, he continued to complain of episodes of temporary worsening of limb weakness 2 to 3 times a year lasting for several days. He could identify no specific triggers. At the age of 38 years, he presented with an episode of severe neck flexor and bulbar weakness. Over the course of 9 days, his neck flexor weakness improved to baseline. Bulbar function remained impaired, and a percutaneous endoscopic gastrostomy (PEG) tube for feeding was inserted. However, he continued to report fluctuant improvement in his swallow, on some days being entirely reliant on the PEG and on others managing a soft diet. Fourteen months after the PEG insertion, he attended clinic and reported that he no longer used it and consistently managed a normal diet. Videofluoroscopy demonstrated only minor persisting abnormalities, and the PEG was removed at his request.

**Figure 2:**
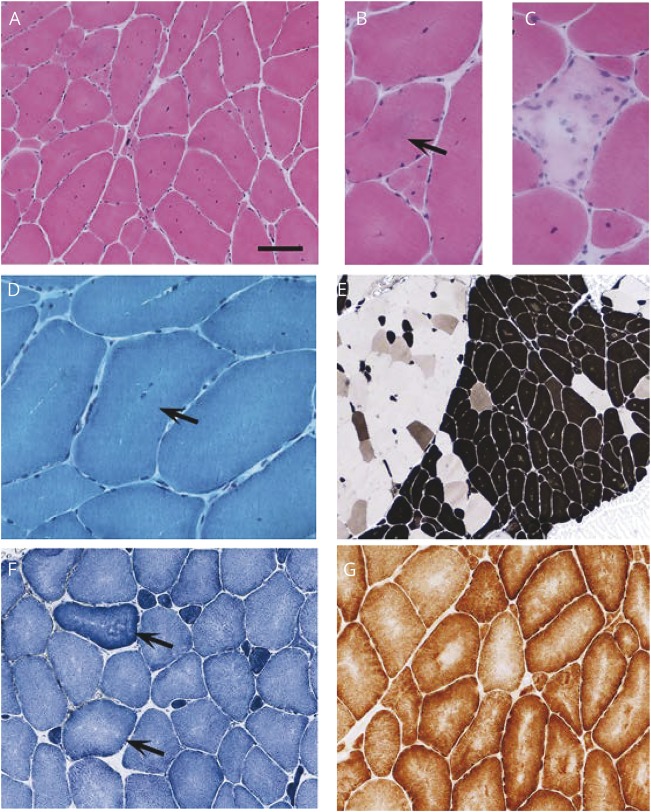
Histologic examination of a right biceps muscle biopsy Histologic examination of a right biceps muscle biopsy performed at age 42 years showed (A) variation in fiber diameter and increased internal nuclei with (B) occasional fibers showing central basophilia (arrow) and (C) a small number of necrotic fibers.Gomori trichrome staining suggested regions with reduced mitochondrial staining (arrow). (E) ATPase histochemistry indicated focal type I fiber predominance (darkly stained fibers). (F) Mild central pallor suggestive of cores was apparent in type I and II fibers in the nicotinamide adenine dinucleotide tetrazolium reductase preparation (arrows), and (G) central reduction in oxidative enzyme activity was confirmed by cytochrome oxidase histochemistry. Scale bar in represents 260 μm in (E); 100 μm in (A), (F), and (G); and 50 μm in (B–D).

Over subsequent years, he continued to experience acute attacks of myalgia followed by weakness. When care was transferred to us, he was reinvestigated, including a repeat muscle biopsy at the age of 42 years (figure 2), which on this occasion revealed mild loss of oxidative enzyme activity in the central region of many fibers, but classic central cores were not observed.

Electrophysiologic tests showed myopathic EMG features, normal repetitive stimulation, mildly abnormal jitter, and occasional blocking on single-fiber EMG (including in extensor digitorum communis, biceps, and orbicularis oculi). While indicative of a mild neuromuscular junction transmission defect, this was thought likely to be a secondary phenomenon rather than a primary myasthenic feature.^13^ Serologic testing for acetylcholine receptor antibodies and muscle-specific kinase antibodies was negative. The McManis test for PP was positive on 2 separate occasions (53% and 59% decrement in compound muscle action potential [CMAP] amplitude). MRI demonstrated fatty infiltration of all thigh muscles, in particular the adductor magnus, which was almost completely replaced by fat, with relative sparing of the vastus lateralis, rectus femoris, gracilis, and semitendinosus, a pattern of selective involvement previously associated with *RYR1*-related myopathies.^14,15^ There was no family history of similar symptoms. Genetic analysis revealed *RYR1* variants Arg109Trp, previously described with MmD and ophthalmoplegia,^4,16^ a variant of uncertain significance Met485Val,^4^ and the novel although presumed truncating variant Gln70X. Testing for congenital myasthenia and PP genes was negative. His unaffected mother carried the Gln70X variant. It was not possible to obtain DNA from his father.

### Case 2

A 42-year-old Swiss woman presented with intermittent weakness of the limbs lasting from several minutes to 2 days. Medical history was notable for migraine and 3 caesarean sections. The first episode of flaccid paresis occurred during her first pregnancy at the age of 23 years with a fall from the couch when she could not move her right arm and leg for several minutes, without associated disturbance of cognition or sensory symptoms. Examination at the emergency room after recovery was normal. Brain imaging was not performed because of the pregnancy. Two subsequent EEGs were normal, and no specific diagnosis was made at the time. She had a history of migraine, and we cannot definitively exclude the possibility that this first episode of unilateral weakness was a migraine aura without headache. However, her typical migraine episodes are headache and are distinct from this presentation. Fifteen years later, while driving, she noted weakness of her arms, legs, and trunk severe enough to warrant stopping the car. Weakness recovered slowly after 15 minutes. A similar episode occurred a few months later. Potassium levels and clinical examination when asymptomatic were normal. Subsequently, the frequency of similar episodes with flaccid paresis of her limbs, affecting predominantly the legs and lasting several minutes, increased and they occurred daily, always after resting. No correlation with food intake or fasting was reported. In addition, she complained of painless cramps in her arms and legs muscles, which could become painful if she tried to stretch her muscles. These lasted up to 10 minutes and also occurred during sleep.

A detailed neurologic examination at the age of 42 years revealed no abnormalities. A relatively thin and long face with a small lower jaw was noted, but she was not overtly dysmorphic. On follow-up examination, she presented with flaccid weakness of her right hand lasting for 2 days. Motor and sensory nerve conduction studies, repetitive motor nerve stimulation, and EMG in the limbs and paravertebral muscles performed after recovery from the acute episode were unremarkable. The McManis test was negative. Laboratory testing revealed no thyroid dysfunction; electrolytes and creatine kinase were normal. Muscle biopsy of the tibialis anterior muscle was unremarkable. Genetic testing revealed *RYR1* gene variants Arg1507Gln and Gly2446Ser in trans*.* Arg1507Gln is a missense variant with a minor allele frequency of 0.00001 in ExAC that has been previously found in other myopathic phenotypes. Gly2446Ser, although not previously reported, localizes to a recognized MH-associated mutational hot spot (www.emhg.org). PP gene testing was negative.

### Case 3

This 49-year-old man reported experiencing episodes of minor limb weakness after strenuous exercise that lasted a few hours from the age of 14 years. He had his first full attack of muscle paralysis at the age of 29 years. He awoke in the morning after a day of strenuous exercise to find that he was unable to walk. From this age on, he experienced multiple similar episodes. All muscles from the neck down could be weak, although the legs were predominantly and most severely affected. Symptoms usually lasted for several hours but could persist for as long as 48 hours. In the recovery phase, he reported that the limbs could be painful as strength returned. Symptoms could be provoked by exercise, intense heat, or a carbohydrate-rich meal late at night.

Medical history was notable for 2 complicated general anesthetics in childhood, although the exact circumstances were unclear and medical records unavailable. His parents were advised after these that he may be at risk of MH and to inform clinicians before any future procedures requiring general anesthesia.

Examination was unremarkable, as was creatine kinase and MRI of the lower limbs. EMG and nerve conduction study were normal, but the McManis test for PP was positive with an exercise-induced reduction of CMAP of 68%. A biopsy of the tibialis anterior muscle demonstrated variation in fiber size, an increase in internal nuclei, and 1 ring fiber (figure e-1, http://links.lww.com/WNL/A96). A type I fiber predominance was interpreted as normal for the muscle biopsied. Genetic testing for PP genes was negative, but an *RYR1* variant Arg1043His was identified. Another amino acid substitution at the same site, Arg1043Cys, previously associated with the MH trait but not functionally tested yet (www.emhg.org), has been described.

## Discussion

We have previously reported a single patient with recessive *RYR1*-related MmD with additional episodic muscle weakness/paralysis.^11^ Here, we have described 3 additional cases (summarized in the table). One was compound heterozygous for *RYR1* nonsense and missense mutations and presented with later development of episodic symptoms in the context of a congenital myopathy, similar to our original patient reported by Zhou et al.^17^ who showed a comparable genotype and phenotype. The other 2 patients had an episodic phenotype without myopathy and were either heterozygous for or compound heterozygous for *RYR1* missense mutations (putatively) implicated in the MH trait, suggesting tentative genotype-phenotype correlations. In all cases (including our original case), the onset of episodic muscle paralysis was from early adulthood onward, which is later than the onset typically seen in primary PP.^17^ Although there were variable triggers, including exertion or rest after exertion, which is a typical trigger for PP, there was no consistent relationship to food or temperature across all the cases. Myalgia or cramp was a universal feature, indicating overlap with the spectrum of *RYR1*-related exertional myalgia/rhabdomyolysis, and this, together with the later age at onset, may be a phenotypic clue for future cases.

**Table:**
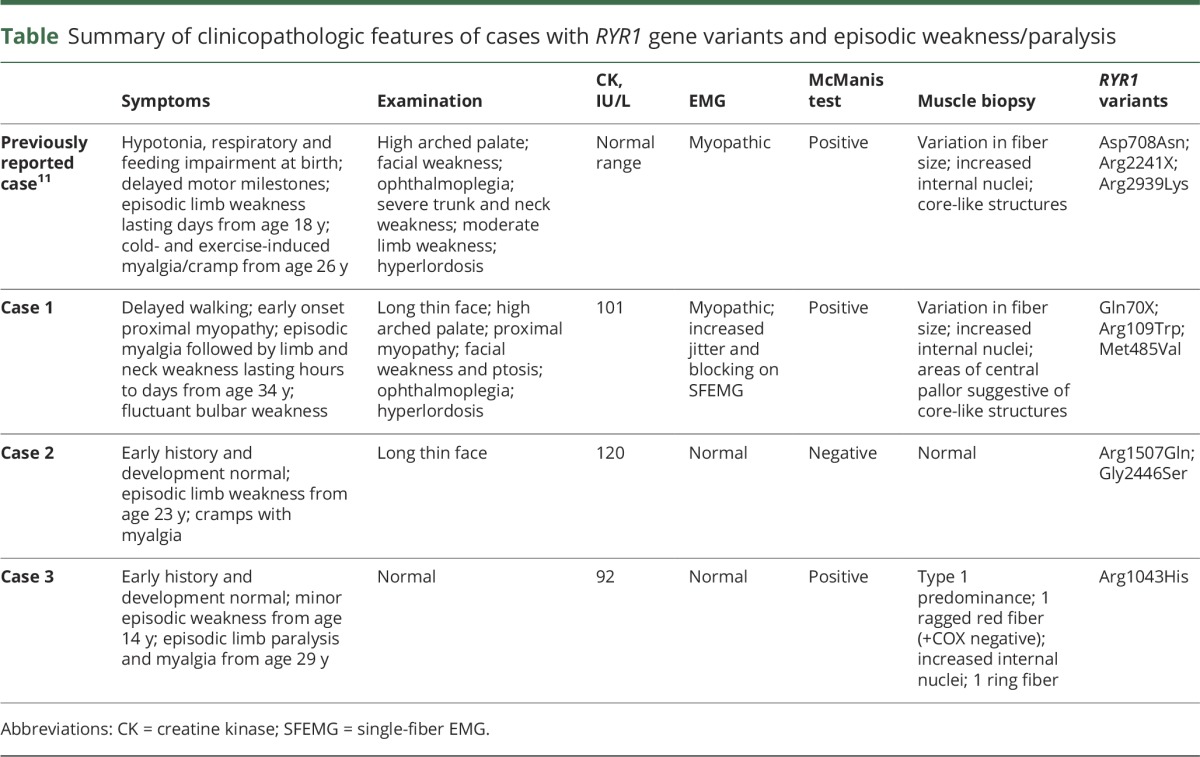
Summary of clinicopathologic features of cases with *RYR1* gene variants and episodic weakness/paralysis

The McManis test was positive in 2 of 3 of our new cases. We retrospectively reviewed the source data for our previously reported case^11^ and found that the drop in CMAP amplitude when calculated from the postexercise increment was also >40%, indicating a positive test.

A positive McManis test, to the best of our knowledge, has previously been described only in primary or secondary PP.^18^ Given the direct interaction between the Cav1.1 channel implicated in the majority of cases of hypokalemic PP^19^ and *RYR1*, it seems plausible that dysfunction of each may produce phenotypic mimics. This is supported by the observation of a profound disturbance of the normal Cav1.1/*RYR1* interaction in 1 previous patient with *RYR1*-related PP and other patients with recessive *RYR1*-related myopathies.^11,20^ Furthermore, the recently described *CACNA1S*-associated early-onset myopathy shares features of predominantly axial weakness and ophthalmoplegia with *RYR1*-related myopathies^12^ and may also feature similar reductions of the Cav1.1 protein. However, the exact mechanism by which the *RYR1* variants would cause an episodically unexcitable sarcolemma as demonstrated by a positive McManis test is unclear. On the basis of the recent observation of altered BK channel activity in RYR1-mutated smooth muscle cells,^10^ one possibility is via an impairment of BK channel–mediated membrane repolarization due to altered intracellular calcium homeostasis, but this requires further study.^10,21,22^

Other atypical PP phenotypes associated with motor neuropathy due to mitochondrial gene mutations have been described,^23^ demonstrating that the clinical symptoms may be seen in non–ion channel genetic disorders. Our data demonstrate that late-onset episodic muscle weakness or paralysis with prominent myalgia and cramps may be a more common *RYR1*-associated phenotype than the single case we previously reported and may not be limited to a specific genotype. We suggest that cases compound heterozygous for *RYR1* nonsense and missense mutations are more likely to have a concomitant early-onset myopathy. The McManis test may be a useful diagnostic tool in these cases, although a negative test does not exclude the possibility of *RYR1* involvement.

We propose that clinicopathologic features suggestive of *RYR1* disorders should be sought in genetically undefined PP cases and *RYR1* gene testing considered in those in whom mutations in *SCN4A, CACNA1S*, and *KCNJ2* have already been excluded.

## Glossary

CAMP: compound muscle action potential
MH: malignant hyperthermia
MmD: multiminicore disease
PEG: percutaneous endoscopic gastrostomy
PP: periodic paralysis
RYR1: ryanodine receptor
